# Radon Mitigation Approach in a Laboratory Measurement Room

**DOI:** 10.3390/s17051090

**Published:** 2017-05-11

**Authors:** Patricia Blanco-Rodríguez, Luis Alfonso Fernández-Serantes, Alberto Otero-Pazos, José Luis Calvo-Rolle, Francisco Javier de Cos Juez

**Affiliations:** 1 Department of Energy, University of Oviedo, Oviedo 33004, Spain; blancopatricia@uniovi.es; 2 Department of Industrial Engineering, Universidade da Coruña, Ferrol 15405, Spain; luis.alfonso.fernandez.serantes@udc.es (L.A.F.-S.); alberto.otero.pazos@udc.es (A.O.-P.); 3 Department of Mines Exploitation and Prospecting, University of Oviedo, Oviedo 33004, Spain; fjcos@uniovi.es

**Keywords:** radon, mitigation, positive ventilation, sump system, sealing, radon reduction

## Abstract

Radon gas is the second leading cause of lung cancer, causing thousands of deaths annually. It can be a problem for people or animals in houses, workplaces, schools or any building. Therefore, its mitigation has become essential to avoid health problems and to prevent radon from interfering in radioactive measurements. This study describes the implementation of radon mitigation systems at a radioactivity laboratory in order to reduce interferences in the different works carried out. A large set of radon concentration samples is obtained from measurements at the laboratory. While several mitigation methods were taken into account, the final applied solution is explained in detail, obtaining thus very good results by reducing the radon concentration by 76%.

## 1. Introduction

Radon is a gas produced by the natural radioactive decay of $U^{238}$, a very active chemical element capable of associating with many others and moving with them [1]. Uranium is present in small proportions (ppm) in almost all rocks and soils of the planet [2]. Galicia Autonomy (Spain) is an area prone to radon gas due to the common presence of granites, a type of rock and soil that has great uranium concentration [3,4].

Radon is a naturally produced inert radioactive gas. It is odorless, colorless and tasteless [5]. It is easily released from the soil into the air, where it disintegrates forming various short-lived products known as radon progeny [6]. In the decay mode of radon gas, alpha particles are emitted, which adhere to aerosols, dust and other airborne particles [7]. As a consequence, when people breathe, radon progeny is deposited on epithelial cells lining the airways where alpha particles can damage DNA and, therefore, cause lung cancer [8] and thousands of deaths all over the world each year [9]. According to the United States Environmental Protection Agency (EPA), radon gas is the second leading cause of lung cancer, after smoking, causing 21 thousand deaths per year in the United States [10].

Due to its accumulation, radon can be a severe issue in houses, workplaces, schools or any building where people or animals are located. It comes from the soil basement where buildings are located and it enters them through cracks in the floors, construction joints, gaps in suspended floors, cavities inside walls or water supply.

Normally, radon gas is not produced by common building materials [9] and, as a general rule, outdoor radon concentration is very low. On average, it varies between 5 and 15 Bq/m^3^ [7]. Conversely, indoor concentrations are higher and reach maximums at places such as mines, caves and treatment water plants, among others [7].

Like in other fields [11], Spanish legislation establishes that the average concentration of radon at workplaces should be less than 600 Bq/m^3^ (300 Bq/m^3^ for workplaces with high permanence of workers). Over this value (>600 Bq/m^3^), a mitigation control system with periodical tests should be installed to keep radon levels low [13].

The equipment used for environmental radioactivity measurements is very sensitive and needs specific conditions, especially regarding temperature, pressure and relative humidity. Consequently, it is necessary to achieve optimal operating conditions and, therefore, there should be no presence of perturbation or any radioactive contamination such as radon gas concentration in the place where the equipment is located. Significant radon gas levels will involve an undesirable increase in the variability of measurements. Of course, this should not occur if very precise measures are required.

There are some studies about radon mitigation in the literature, mainly in households; however, there are no cases of laboratory measurement rooms. This research shows a specific study of this last case, where an initial analysis and monitoring of radon gas concentration was made with common equipment. Then, consequent mitigation solutions were checked and implanted for the required reduction, providing specific data during operation for the adopted solutions. It should also be taken into consideration that the laboratory location is in a geographical area with high radon gas concentrations due to the soil nature, which significantly complicates the main goal.

The use of a high precision sensor under a strictly controlled scenario enables the validation of a certain set of assumptions neither verified nor published so far.

Different factors play an important role in decreasing radon gas concentration when the number of air changes per hour in a room is above four. This is of particular importance in laboratories equipped with environmental radioactivity measurement devices, each one of them having its own sensor. These specific measurement rooms are key to controlling environmental radioactivity, a vital issue both at national and European level.

The EU Member States have a collective responsibility to ensure continuous monitoring of radioactivity in the air, water and soil, in accordance with Articles 35 and 36 of the Euratom Treaty concerning monitoring of radioactivity in the environment. Additional EU legislation sets out detailed criteria for the quality of water intended for human consumption, and the maximum permitted levels of radioactive contamination of foodstuffs (Directive 98/83/EC and Regulation 733/2008/EC respectively).

Reliability of the environmental radioactivity equipment measurements largely depends on the mitigation methods adopted to reduce radon gas concentration. As a consequence, in a critical area such as environmental radioactivity monitoring, it is essential not only to include a contrast methodology but also to establish a clear configuration of the measurement room and its equipment.

In order to reduce radon gas concentration at workplaces, several mitigation solutions can be implemented, such as:

- Sealing: trying to seal all obvious cracks, gaps and holes in floors or walls to prevent radon from entering the building. However, in most cases, reduction is not effective [14,15].
- Positive ventilation: installing small fans to blow filtered fresh air into the building. The fundamental principle is to increase the pressure inside the building in relation with outside pressure, so radon gas cannot flow inside [4,14,15].
- Sump systems: the purpose is to discharge the air from inside the building when the concentration reaches a set point value. Usually, the extraction is forced to improve the performance and to reduce the response time [15,16,17].
- Underfloor ventilation: installing vents at the foundations to provide natural or force ventilation underfloor to suck or blow radon [4,15].

The proposed solutions in the present work are based on positive ventilation and their main aim is to avoid interferences with the measurement equipment at the radioactivity laboratory. This study is focused on the analysis of different steps that have been carried out in the mentioned case. For each step, a change has been introduced and several measurements have been done. Finally, a comparison was made, taking into account all recorded cases.

This paper begins with a description of the case study, where both the installation and all the different components used to decrease radon concentration are detailed. It follows with the carried out process that analyses the approach, step by step. Then, the results are shown and finally the conclusions are drawn.

## 2. Materials and Methodology

In this section, the specific radiation monitoring sensor as well as the followed methodology are described.

### 2.1. Materials

The sensor employed to measure radon gas activity is the AlphaGUARD PQ 2000PRO model from SAPHYMO [18].

AlphaGUARD PQ 2000PRO is a portable device used for instantaneous or continuous radon concentration measurements, both for short- or long-term tests in buildings and outdoors.

This sensor, which is insensitive to vibrations as well as humidity, has a pulse-counting ionization chamber (alpha spectroscopy) and allows radon gas levels to be measured in a wide range, between 2 and 2, 000, 000 Bq/m^3^. Operating in diffusion mode, the user can select measuring cycles from 10 min to 60 min, while operating in flow-through mode the cycle time varies between 1 and 10 min.

Among its many advantages, AlphaGUARD PQ 2000PRO performs fast response with high detection efficiency (1 cpm at 20 Bq/m^3^), and enables maintenance-free operation with a stable calibration. The instrument calibration error amounts to 3%, plus uncertainty of the primary standard.

In addition to the radon gas concentration, AlphaGUARD PQ 2000PRO is equipped with different integrated sensors that enable other essential parameters in radon mitigation to be measured and stored, such as ambient temperature, atmospheric pressure and relative humidity. The internal sensor included to measure ambient temperature, from −10 °C to 60 °C, is a precision monolithic integrated circuit. The device also has a laser-trimmed silicon bridge transducer in order to determine atmospheric air pressure, from 800 mbar to 1050 mbar. Finally, the integrated sensor that allows to measure relative humidity, from 0% rH to 99% rH, is an hydrophilic polymer film on hybrid.

A specific software called DataEXPERT, included together with the AlphaGUARD Radon Monitor in the basic configuration sold by company Saphymo GmbH, was used for storing and analyzing these data.

The main technical characteristics of the AlphaGUARD PQ 2000PRO sensor are provided below (Table 1).

### 2.2. Methodology

The AlphaGUARD PQ 2000PRO detector is based on a design-optimized pulse ionization chamber. In regular operation, the measuring gas enters diffusion mode through a large-surface glass fiber filter into the ionization chamber. In addition, the glass fiber filter protects the interior of the chamber and thus prevents contamination of dusty particles. While the radon progeny products do not enter the ionization chamber, only the gaseous Radon-222 reaches the chamber passing through the glass fiber filter.

For our purpose, the AlphaGUARD sensor operates in diffusion mode measuring radon gas concentration every 10 min. With the obtained values, the equipment calculates the average as in Equation (1).

(1) $$Average=\frac{1}{n}\cdot \sum_{i=1}^{n}x_{i}$$

It also calculates the uncertainty as the standard deviation using the Equation (2).

(2) $$Uncertainty=\sqrt{\frac{\sum_{i=1}^{n}{\left(x_{i}-\bar{x}\right)}^{2}}{\left(n-1\right)}}$$

## 3. Case Study

The Environmental Radioactive Laboratory of the University of A Coruña (Spain) carries out different works related to radioactive measurements and the analysis of the obtained data. These tasks are carried out in a specific room (measurement room), where all equipment used in this study is located. The case study is described in detail in the following subsections.

### 3.1. Environmental Radioactive Laboratory

The proposal has been implemented at the *“Laboratorio de Radiactividad Ambiental”* (LRA) of the University of A Coruña, located at the Faculty of Engineering in Ferrol (Galicia Autonomy). The laboratory carries out different studies on the radiological characterization of environmental samples on the north coast of Galicia or with a specific agreement with the Nuclear Safety Council, on a program of Environmental Radiation Monitoring, for example.

The characterization measurements of samples are done in a small room, where a concentration of radon gas has been detected. This laboratory measurement room was chosen because it is a small room, closed and with little ventilation, an appropriate place where radon gas concentrations can be high.

The radon level was first measured in the laboratory measurement room. Because radon gas is present in detectable concentrations in this specific laboratory, it was chosen to be the site to study different mitigation methods. Some of these methods were implemented with the consequent reduction of radon level.

### 3.2. Laboratory Measurement Room

**Figure 1:**
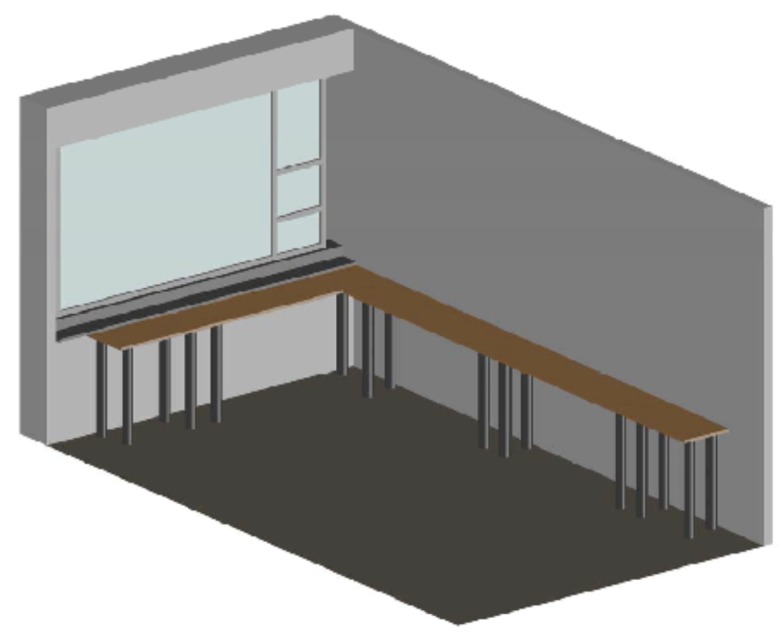
Measurement room.

The LRA measurement room is a small room of 20 × 60 m^2^ where all radioactive measurements are done and where the used equipment is placed. The room is located inside the LRA, on the second floor of the building. It is west-oriented and its dimensions are, approximately, 3.20 m high, 5.80 m long and 3.55 m wide. Figure 1 shows the overall appearance of the room.

The building structure and the floor are made of reinforced concrete with the walls made of coated and painted bricks. Due to the heavy weight of the equipment, it was necessary to install steel sheets on the floor in order to distribute the weight uniformly. To get inside this room, there is a door that connects with the chemistry laboratory and the office. Moreover, there is a window situated on the west wall where the different mitigation systems are installed.

## 4. Radon Mitigation Solution Approach

With the aim of reducing radon gas concentration and avoiding perturbations in the equipment, several mitigation methods have been taken into account. The implemented solution had to consider building limitations and restrictions. On the other hand, due to the fact that the measurement room is independent of the office, the applied solution does not disturb the work environment.

The measurement room is located on the second floor and, as a result, some mitigation methods such as underfloor ventilation are impossible to carry out. In addition, sealing and sump systems cannot be implemented because of the building age and because there are different entrances to the room. In any case, radon gas would have entered the measurement room through the cracks.

Instead, positive ventilation could be a good solution because of its viability and low-cost implementation and, due to the reasons described above, this is the chosen solution to mitigate radon.

### 4.1. Positive Ventilation Solution

Positive ventilation consists of fans that blow fresh and filtered air into the measurement room, by pressurizing to prevent radon from entering and diluting it [14,15]. To implement the selected solution, two fans and an air conditioning system were installed. Different steps were followed to carried out this option.

The fans were installed in order to pressurize and dilute radon gas. Installation of two fans was found to be necessary to provide enough ventilation.

Besides, an air conditioning system was installed to reduce air humidity, keep the temperature around the value of 20 Celsius degrees and to weatherize the measurement room. Moreover, this system blows filtered air into the measurement room, contributing to radon mitigation and its stability throughout the year. Air conditioning was necessary to ensure constant environmental conditions.

These systems also benefit the installation because they reduce condensation.

**Figure 2:**
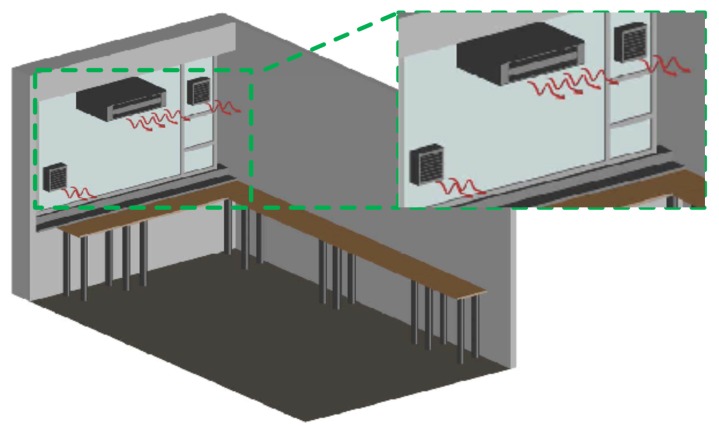
Measurement room with all systems.

They were installed at the unique window of the room, so the works carried out for their installation were minimal. Figure 2 shows the measurement room with the installed systems.

The components used to decrease radon concentration are the following:

- Fans: The implemented fans are the HV-230 model from S&P, whose characteristics are:
  - Rotating speed of 1250 revolutions per second.
  - Extraction caudal at high speed of 600 m^3^/h and at low speed of 450 m^3^/h.
  - Impulsion caudal of 330 m^3^/h.
  The power consumed by the fans amounts to 34 watts each, and the sourced current is 0.15 amperes.
- Air conditioning: The air conditioning system is the PCA-RP50xB7KA model from Mitsubishi Electric, whose characteristics are:
  - For cold, 5 kW and 4300 kCal/h.
  - For hot, 5.5 kW and 4730 kCal/h.
  - Sound level, for low speed, around 32/40 dB.

### 4.2. Steps Followed until the Final Solution is Reached

**Figure 3:**
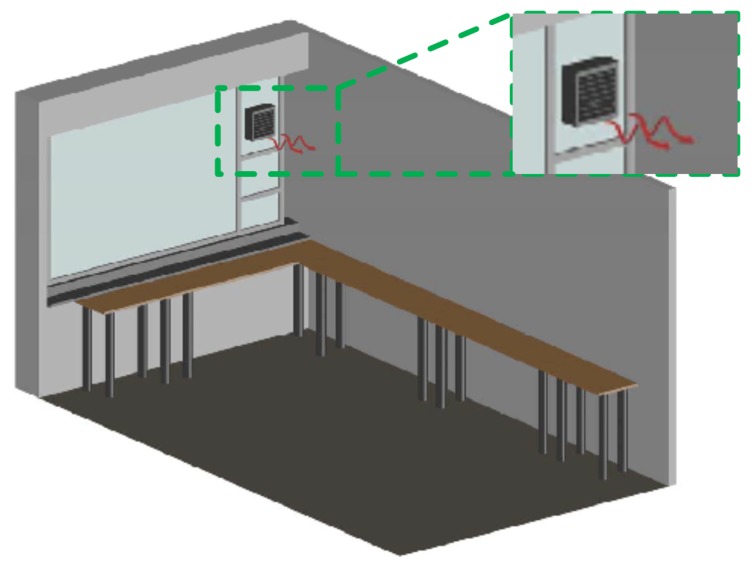
Measurement room with one fan.

**Figure 4:**
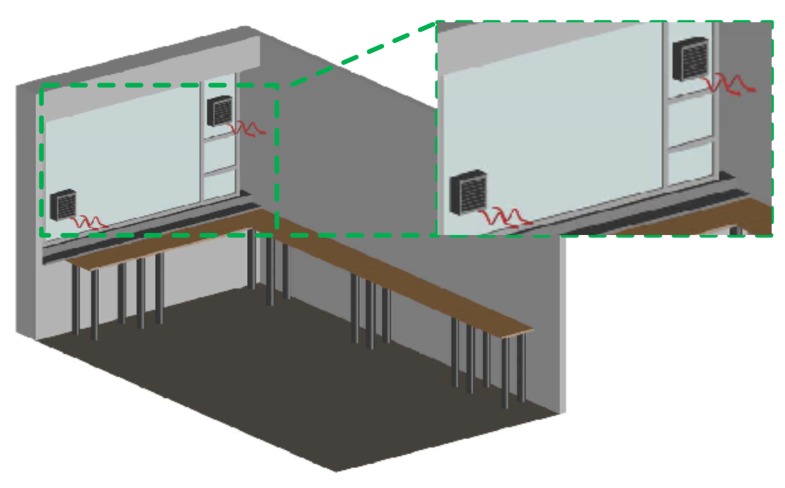
Measurement room with two fans.

In this subsection, the steps carried out to obtain the desired results are explained:

1. The starting point was the installation of one fan to pressurize the measurement room, see Figure 3. Radon concentration was measured some time later, once its level had achieved a steady state, but the level did not decrease enough with this action.
2. As a result, a second fan was installed to increase the pressure inside the measurement room, see Figure 4. After several measurements, it was noticed that the introduced air was sufficient to reduce radon concentrations, thus avoiding interferences in the measurements.
3. Once the fan systems were implemented, air conditioning was installed, see Figure 2. Furthermore, it blows air inside the measurement room and weatherized it at a constant condition at all times. The air conditioning helps to have stable measurements and, therefore, minimize the uncertainty due to the temperature control of the room and the reduction in humidity.
4. In the end, all systems—fans and air conditioning—were working together with the aim to decrease radon concentration and to have repeatable conditions for the measurements. In addition, different analyses were done periodically to keep control of radon levels in order to know how they vary throughout the year.

In this particular case, fans work in impulsion mode and the air conditioning works keeping the temperature around the value of 20 °C and controlling air humidity.

## 5. Results

In this section, the radon concentration measurements for the different cases mentioned above are shown. At first, the radon concentration was measured on different days to quantify its level. Table 2 shows the average concentration for the period from April 25^*th*^ to November 23^*th*^ 2007, without any mitigation solution.

Then, from November 30, 2007, to January 25, 2008, one fan was installed and measurements were carried out. The next period of measuring was from January 25 to December 1, 2008. During this period, a second fan was installed and several measurements were taken with both fans operating. Table 3 shows the concentrations during these periods. The analysis of the concentration while the mitigation solutions were implemented revealed how it decreased to the values shown in Table 3.

After the installation of the two fans, an air conditioning system was installed, achieving the results shown in Table 4. The period when this system (both fans and air conditioning) is on is from 1 December 2008 to nowadays. At this moment, the value of radon concentration is very low. Finally, Table 5 shows the percentage concentration reduction summary by using the different components of the mitigation solution. Remark, in general terms, it is necessary to apply a minimum overpressure of 5 mbar into the measurement room. Nowadays, both fans are always in operation to achieve this value.

**Figure 5:**
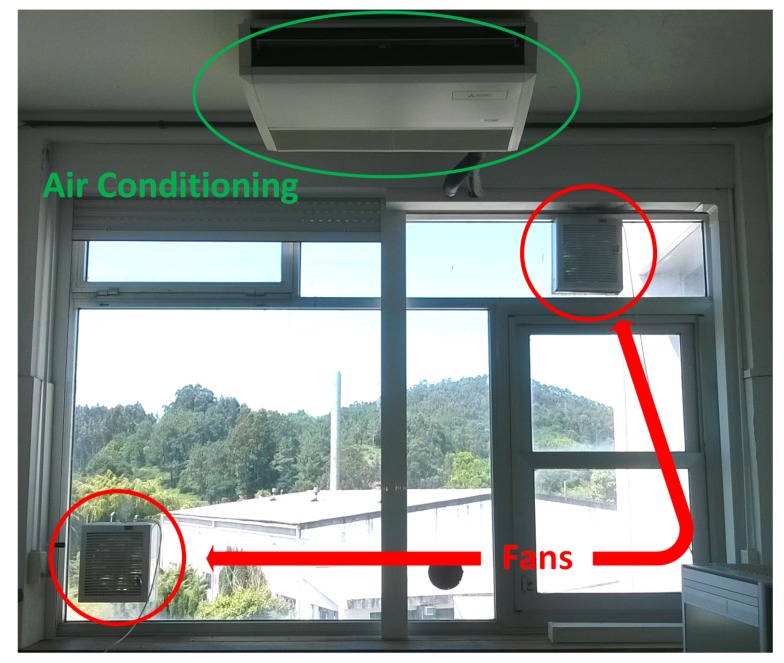
Picture of the measurement room with all systems.

**Figure 6:**
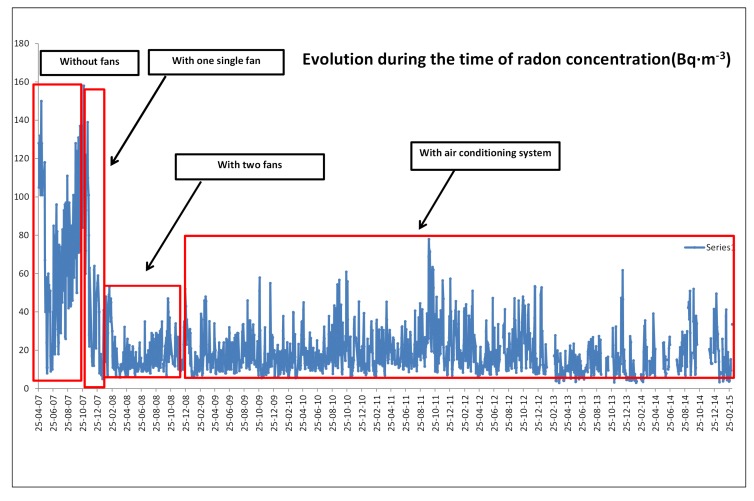
Evolution of radon gas measurement with the different systems.

While Figure 5 shows the measurement room with all the installed systems, two fans and the air conditioning system, Figure 6 shows a summary of the reduction of the radon gas with the different systems, showing how it is reduced when the methods are implemented.

## 6. Conclusions

With the radon mitigation solution approach explained in this paper, very good results have been obtained. As it is shown in the results section, after the installation of different removing systems, the radon concentration decreased in a very significant way, up to the value of 76%. As a consequence, the implemented solution avoids interferences in the laboratory measurements.

Positive ventilation achieved good results by pressurizing the measurement room and avoiding radon entrance. For this purpose, two fans were installed with the aim of reducing the radon concentration to a low value. In addition, air conditioning contributed slightly to radon mitigation and allowed a very good repeatable condition performance for the measurements.

## Figures and Tables

**Table 1:** AlphaGUARD PQ 200PRO characteristics.

**Physical Characteristics**	
Measurement range (Rn-222)	
-Lower limit	2 (Bq/m^3^)
-Upper limit	2, 000, 000 (Bq/m^3^)
Foldback protection	>10,000,000 (Bq/m^3^) verified
Type of radon detector	Ionization chamber (HV=750VDC)
Operation mode	3D alpha spectroscopy and current mode
Detector volume	
-Total	0.62 liter
-Active	0.56 liter
Type of Rn-FP filter	Glass fiber filter
(detector entry window)	(retension coefficient > 99,9 %)
Detector filling mechanisms	Design optimized for fast passive diffusion (10/60 min cycle)
	Flow mode (1/10 min cycle) Rn/Tn mode (10 min cycle)
Detector signal acquisition	Fast digital signal sampling network, using three separate
	ADC-channels
Calibration error (Rn-222)	±3%
Detector efficiency	1 cpm at 20 (Bq/m^3^)
Background signal due to	<1 (Bq/m^3^)
detector contamination	
Measurement cycle time	
-Diffusion mode	10 min or 60 min (user selectable)
-Flow-through mode	1 min or 10 min (user selectable)
Storage capacity	3 days at 1 min measuring cycles
	1 month at 10 min measuring cycles
	6 months at 60 min measuring cycles
Internal sensors	
-Ambient temperature	Precision monolithic integrated circuit (−15 °C–60 °C)
-Atmospheric air pressure	Laser-trimmed silicon bridge transducer (800 mbar–1050 mbar)
-Relative air humidity	Hydrophilic polymer film on hybrid (0%∼99%rH)
**Environmental Characteristics**	
System operating range	
-Operating temperature	−10 °C–50 °C
-Air pressure	700 mbar–1150 mbar
-Relative humidity	0%∼95%
**Mechanical Characteristics and Display**	
Dimensions (excluding handle)	120 mm × 315 mm × 175 mm (H × W × D)
Weight	4.5 kg
**Electrical Characteristics**	
Power supply	Battery or external mains (120 V–230 V)
Battery	Rechargable, allows > 10 days autonomous operation,
	>30 days using external booster
**Interfaces**	
DataEXPERT software for data collection, management and professional analysis	

**Table 2:** Concentration before applying any solution.

Radon-222 Activity	Concentration (Bq/m^3^)
Average	79
Uncertainty	37
Maximum concentration	158
Number of measurements	212

**Table 3:** Concentration after installation of fans.

Radon-222 Activity	One Fan (Bq/m^3^)	Two Fans (Bq/m^3^)	One/Two Fans (Bq/m^3^)
Average	29	19	21
Uncertainty	18	10	13
Maximum concentration	64	53	64
Number of measurements	56	278	334

**Table 4:** Concentration after air conditioning installation.

Radon-222 Activity	Concentration (Bq/m^3^)
Average	19
Uncertainty	9
Maximum concentration	40
Number of measurements	1174

**Table 5:** Concentration reduction after mitigation systems installation.

Radon-222 Activity	Mitigation Solution	Compared with	Reduction %
Average	One fan	Any method	62.8
Uncertainty	One fan	Any method	50.3
Average	Two fans	One fan	35.6
Uncertainty	Two fans	One fan	43.7
Average	Two fans	Any method	76.0
Uncertainty	Two fans	Any method	72.0
Average	One or two fans	Any method	73.8
Uncertainty	One or two fans	Any method	65.8
Average	Two fans and air conditioning	Any method	76.0
Uncertainty	Two fans and air conditioning	Any method	75.7
